# Evaluation of 4-Aminoquinoline Hydrazone Analogues as Potential Leads for Drug-Resistant Malaria

**DOI:** 10.3390/molecules28186471

**Published:** 2023-09-06

**Authors:** Rachael N. Magwaza, Muna Abubaker, Buthaina Hussain, Michael Haley, Kevin Couper, Sally Freeman, Niroshini J. Nirmalan

**Affiliations:** 1 Division of Pharmacy and Optometry, School of Health Sciences, University of Manchester, Manchester M13 9PT, UK; r.n.magwaza@salford.ac.uk; 2 School of Science, Engineering and Environment, University of Salford, Manchester M5 4WT, UK; m.abubaker2@salford.ac.uk; 3 Faculty of Pharmacy, Al-Zaytoonah University of Jordan, Amman 17138, Jordan; buthina.hussein@zuj.edu.jo; 4 School of Biological Sciences, Lydia Becker Institute of Immunology and Infection, University of Manchester, Manchester M13 9PT, UK; michael.haley@manchester.ac.uk (M.H.); kevin.couper@manchester.ac.uk (K.C.)

**Keywords:** 4-aminoquinoline, hydrazone, antimalarial, NQO2 inhibitors, antimalarial drug interaction, SAR

## Abstract

The emergence of resistance to first-line antimalarial drugs calls for the development of new therapies for drug-resistant malaria. The efficacy of quinoline-based antimalarial drugs has prompted the development of novel quinolines. A panel of 4-aminoquinoline hydrazone analogues were tested on the multidrug-resistant K1 strain of *Plasmodium falciparum*: IC_50_ values after a 48 h cycle ranged from 0.60 to 49 µM, while the 72 h cycle ranged from 0.026 to 0.219 μM. Time-course assays were carried out to define the activity of the lead compounds, which inhibited over 50% growth in 24 h and 90% growth in 72 h. Cytotoxicity assays with HepG2 cells showed IC_50_ values of 0.87–11.1 μM, whereas in MDBK cells, IC_50_ values ranged from 1.66 to 11.7 μM. High selectivity indices were observed for the lead compounds screened at 72 h on *P. falciparum*. Analyses of stage specificity revealed that the ring stages of the parasite life cycle were most affected. Based on antimalarial efficacy and in vitro safety profiles, lead compound 4-(2-benzylidenehydrazinyl)-6-methoxy-2-methylquinoline **2** was progressed to drug combination studies for the detection of synergism, with a combinatory index of 0.599 at IC_90_ for the combination with artemether, indicating a synergistic antimalarial activity. Compound **2** was screened on different strains of *P. falciparum* (3D7, Dd2), which maintained similar activity to K1, suggesting no cross-resistance between multidrug resistance and sensitive parasite strains. In vivo analysis with **2** showed the suppression of parasitaemia with *P. yoelii* NL (non-lethal)-treated mice (20 mg/kg and 5 mg/kg).

## 1. Introduction

Malaria is a life-threatening disease caused by protozoan parasites of the genus *Plasmodium*. Malaria transmission from human to human naturally occurs through the bite of infected female Anopheles mosquitoes [1]. Malaria remains a major public health threat, with 247 million malaria cases and 619,000 estimated deaths in 2021. Furthermore, an additional 63,000 estimated deaths were recorded due to service disruptions during the COVID-19 pandemic [1]. Although there has been a decline in global malaria deaths, the disease continues to cause significant mortality and morbidity burdens with severe consequences, especially for pregnant women and children in Africa [2]. Human malaria infection is caused by five *Plasmodium* species, *falciparum*, *malaria*, *knowlesi*, *vivax,* and *ovale* [3,4]. Among these species, *P. falciparum* is associated with the most severe and deadly form of malaria and could lead to several life-threatening complications, including anaemia, organ failure, and cerebral malaria [5]. Malaria treatment and prevention currently relies on chemotherapy, vector control, and more recently, approved vaccine (RT, S/AS01) used in selected regions and recommended for children living in areas with moderate to high transmission [6,7]. The vaccine targets the sporozoite stage of the lifecycle, therefore preventing liver infection [8]. Chemotherapy, specifically artemisinin-based combination therapy (ACT), is the first-line treatment option against uncomplicated *P. falciparum* malaria [1]. Although this treatment regime is very effective, there is a growing body of evidence that artemisinin-tolerant *P. falciparum* parasite strains are emerging and consequently threatening the therapeutic utility of the ACT regimen [9,10,11,12,13,14]. Furthermore, current artemisinin treatment is not broadly accessible in some developing countries because of the costs associated with supplying these drugs [15]. Therefore, effective and affordable drugs are urgently needed.

The quinoline scaffold in synthetic and natural compounds has been shown to have a range of pharmacological activities [16,17,18], including antimalarial. The significant impact on malaria morbidity and mortality of the quinoline compound chloroquine in the 1940s fuelled a belief that malaria might be completely eradicated from the world [19]. Following chloroquine resistance, a series of quinoline drugs including amodiaquine, piperaquine, primaquine, and mefloquine were developed and found to be very active against malaria [16,20]. All the quinoline drugs developed possess at least one basic nitrogen (BaN), suggesting the important role of BaN together with other properties in enhancing the antiplasmodial activity and permeability within the malaria parasite [21]. The relevance of novel analogues of quinoline compounds has been unequivocally established, particularly in the context of providing leads with improved pharmacological and toxicological profiles for overcoming chloroquine resistance [22]. 

We previously synthesised and evaluated a series of 4-aminoquinoline hydrazone compounds as human quinone reductase 2 NQO2 inhibitors, a potential therapeutic target in cancer chemotherapy [23]. *P. falciparum* possesses a type II NADH:quinone oxidoreductase known as mitochondrial type II NADH dehydrogenase of *P. falciparum* (*Pf*NDH2), which is structurally dissimilar to NQO2 but catalyses the same reaction, the oxidation of NADH to NAD^+^ and back to the oxidised form using ubiquinone [24]. This constitutes the rationale for the screening of the 4-aminoquinoline hydrazone compounds against *P. falciparum*. Furthermore, a series of quinoline-based hydrazones have been synthesised and tested for their antimalarial properties [25,26]. The compounds have shown remarkable antimalarial activities and inhibited heme to hemozoin formation [25,26]. In this study, we evaluated the antimalarial activity of 4-aminoquinoline hydrazone analogues and presented an in-depth analysis of their activity in vitro and in vivo.

## 2. Results

### 2.1. Determination of IC_50_ Values for the Activity of 4-Aminoquinoline Hydrazones against the K1 Strain of P. falciparum

Sixteen 4-amino-6-methoxy-quinoline hydrazone compounds (see Figure S1, Supplementary Materials for full structures), with methyl and phenyl substituents at the 2-position (R = Me, Ph) and various substituents at R’ (Table 1), were tested for their antimalarial activities. A 1% parasitised culture at the trophozoite stage was treated with the compounds for 48 h at concentrations of 0.003050–200 µM (four-fold serial dilution) against the *P. falciparum* K1 strain, and the growth was measured using the SYBR Green-based plate reader assay. The IC_50_ values of the quinolones ranged from 0.6 to 49 µM (Figure S2 for graphs), with the majority of the methyl-substituted analogues being more active than the phenyl-substituted analogues, supporting a smaller hydrophobic substituent at the 2-position. Compound **9**, with a benzoic acid substituent on the hydrazone group, was inactive, suggesting that a carboxylate anion impedes antimalarial activity. Chloroquine showed an IC_50_ value in line with published data (0.255 ± 0.049 µM) [27]. 

### 2.2. Time-Course Assay

**Figure 1:**
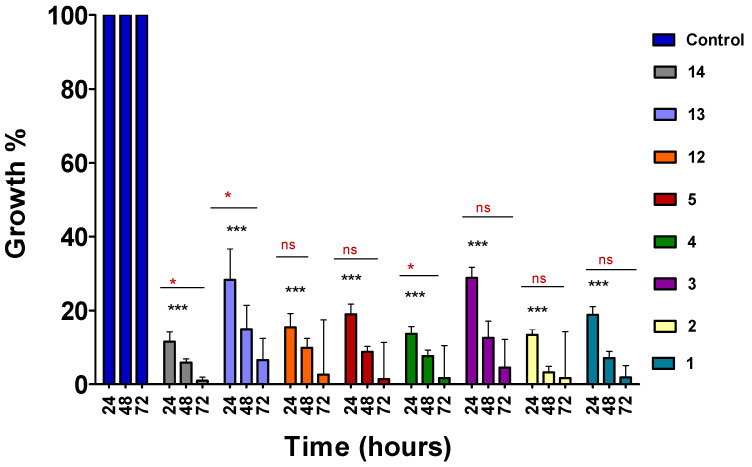
Time-course analysis of 4-aminoquinoline hydrazone compounds. Asynchronous cultures of *P. falciparum* were treated with predetermined IC_50_ doses (Table 1) for 72 h and analysed at 24 h intervals. Drug susceptibility was analysed using the SYBR Green flow cytometer method. Error bars present the standard deviation of two biological replicates. The significant difference between the control vs each compound is indicated by *** *p* < 0.001, while the significant difference between each compound at different time intervals is indicated by ns for *p* > 0.05 and * for *p* < 0.05. The significant difference was determined by two-way ANOVA.

A panel of compounds were selected based on activity and progressed in the time-course assay to define the activity timelines of the compounds. The assay was performed on 1% unsynchronised *P. falciparum* K1 cultures treated with previously determined IC_50_ (Table 1) doses of the lead compounds for 24, 48, and 72 h. Following 24 h incubation, the compounds exhibited activity with a more than 50% reduction in parasitaemia levels, suggesting early onset antimalarial activity (Figure 1). Compounds **3** and **13** showed delayed activity, although they showed better activity on the dose–response assay in comparison to some of the compounds that showed early onset activity. It was also observed that the activity of the compounds became more pronounced as the time progressed up until 72 h of exposure. 

### 2.3. Dose–Response of Lead Compounds after 72 h Incubation Period

The 72 h IC_50_ assay was carried out based on the observation from the time-course assay, which showed that the hydrazone compounds were more active as time progressed. We set up drug efficacy assays for the eight lead compounds **1**–**5**, **12**–**14** (selected based on the best antimalarial activities) (Table 1), with 72 h incubation on parasitised culture. A dose–response experiment was initiated on 1% synchronous culture at the ring stages of the *P. falciparum* K1 strain. The selected compounds were tested at three-fold serial dilutions from 6.86 to 5000 nM. The IC_50_ of most of the compounds after 72 h exposure time were observed in the low nano-molar range ((Table 2), Figure S3 for data), in comparison to the 48 h assay (Table 1). The selectivity indices (IC_50_ human cells/IC_50_ parasites) were calculated based on the 72 h exposure studies and are presented in Table 2. Compounds **1** and **2** were found to be ten-fold more active with high selectivity indices on the MDBK cells. Compounds **4**, and **14** on the other hand showed narrow selectivity indexes, although the anti-*Plasmodium* activity increased significantly. No direct correlation could explain these differences; however when compounds **5** (R = methyl, R’ = 2-hydroxy-3-methoxyphenyl) and **12** (R = phenyl, R’ = 2-hydroxy-3-methoxyphenyl) were compared, it was observed that the substituents on the aromatic ring (R) had a noticeable effect on the cytotoxicity of the compounds, although not much difference was observed on the inhibitory activity of *P. falciparum* parasites.

### 2.4. MTT Cytotoxicity Assay

An MTT (3-(4,5-dimethylthiazol-2-yl)-2,5-diphenyl tetrazolium bromide) assay was performed to investigate the toxicity of the 4-aminoquinoline hydrazone compounds. HepG2 and MDBK cells were incubated with a two-fold serial dilution at dose ranges from 0.39 to 25 µM for 5 days. Cisplatin was used as a control drug to validate the MTT assay. The cell viability data are provided in Table 1. Considering that these compounds were initially designed as anticancer drugs and have shown toxicity on an SKOV-3 ovarian cancer cell line [23], we anticipated that the compounds might be more toxic on the HepG2 cancer cell line. Hence, the compounds were screened on an MDBK cell line, which showed slightly less toxicity. Compound **4** showed a similar toxicity on both HepG2 and MDBK with IC_50_ values of 1.59 μM and 1.66 μM, respectively, while the rest of the compounds showed lower toxicity on the MDBK cells (see Figures S4 and S5). The toxicity of compounds **4**, **7,** and **14** on both HepG2 and MDBK cells, is possibly attributed to the presence of a nitro group in the structures [28].

### 2.5. Stage Specificity Analysis

**Figure 2:**
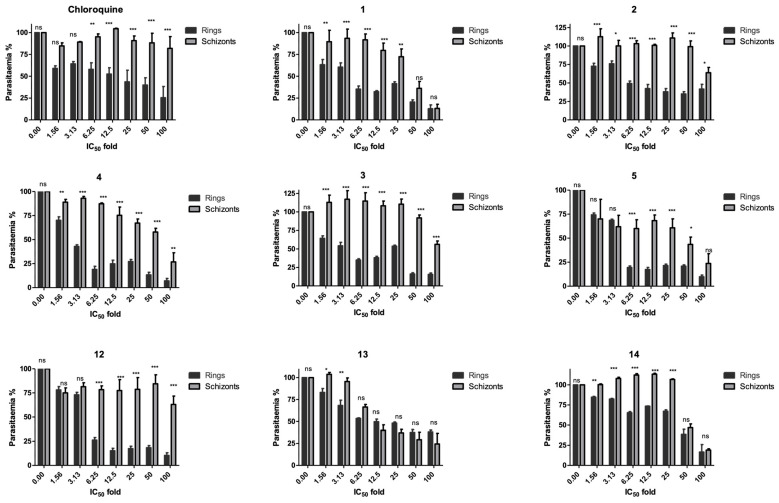
Stage-specific effect of 4-aminoquinoline hydrazone compounds (**1**–**5**, **12**–**14**) on synchronous cultures of *P. falciparum* K1 strain. The drug effects are expressed as a percentage of growth of the respective development stage relative to untreated control. Error bars represent the standard errors of the results from experiments performed three times with each concentration. The significant difference between the ring and schizont stage for each compound at various concentrations is indicated by ns for *p* > 0.05 and * for *p* < 0.05, ** for *p* < 0.01, and *** *p* < 0.001. The significant difference was determined by two-way ANOVA.

The stage specificity was evaluated by conducting drug susceptibility experiments on synchronous cultures either at early rings or schizonts stages (as detailed in the Section 4). Each synchronous culture was treated with 1.6–100 times the respective IC_50_ (Table 1) concentrations of the eight lead compounds (**1**–**5**, **12**–**14**) along with chloroquine as a control. Cultures were exposed to drug treatment for 24 h, after which the compounds were washed out and incubated in complete RPMI medium for a further 24 h. It was observed that all compounds primarily affected the ring stage of the parasite (Figure 2). An effect was observed on the schizont stage for compounds **5** and **14**, inhibiting more than 25% parasitaemia growth at 50–100 times IC_50_. Compound **13** was observed to have a similar effect on both the schizont stage and ring stage. 

### 2.6. In Vitro IC_50_ Values against 3D7 and Dd2 P. falciparum Strains

The lead compounds **1** and **2** were further evaluated against the chloroquine-resistant *P. falciparum* Dd2 strain and chloroquine-sensitive *P. falciparum* 3D7. The drug activity assay was initiated on 1% infected blood at ring stages of the parasites at four-fold serial dilution from 0.00305 to 200 µM. The growth was measured after 72 h using an SYBR Green-based plate reader assay. Both compounds exhibited antiplasmodial activity at lower micro-molar ranges against both strains (Table 3, Figure S6). The results showed that the inhibitory potencies observed for compounds **1** and **2** on the Dd2 multidrug-resistant strain are similar to the sensitive strain 3D7, suggesting no cross-resistance with any of the multidrug-resistant strains tested. When comparing compound **1** in K1 and 3D7 strains, compound **1** was seven-fold more active on the K1 strain.

### 2.7. In Vivo Assay on P. yoelii Mouse Model

**Figure 3:**
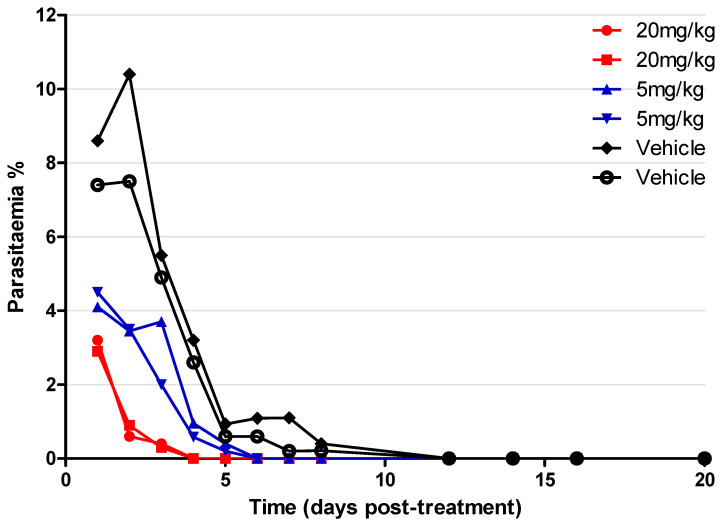
Parasitaemia in infected (*P. yoelii*) mice during and after treatment with compound **2** at 5 mg/kg or 20 mg/kg doses and placebo for 4 days.

We next assessed the antimalarial activity of compound **2** in vivo within a *P. yoelii* NL murine model of blood-stage malaria. The experiment was conducted in a blind mode, where the researcher analysing the parasitaemia was not aware of which group of mice had received the compound or the vehicle. Two dosages of the drug were tested, 5 mg/kg and 20 mg/kg (intravenous tail vein injection), while another group of mice was administered with the drug vehicle alone (Figure 3). The lead compound **2** was injected once a day for 4 days, starting at day 11 when the infection level reached ~5% parasitaemia. Mice were monitored for a total of 20 days in order to detect signs of infection rebound or eventual toxicity. Comparing drug-treated mice to the placebo vehicle control, the parasitaemia of all mice treated with the drug always remained detectable, although, at very low levels, less than 4% after drug treatment was suspended while the parasitaemia in the control increased. There were no detectable signs of significant toxicity or infection relapses as physical or behavioural distress was not detectable in treated mice for over three weeks. 20 mg/kg doses produced almost complete suppression of parasitaemia and cured the mice, while the 5 mg/kg doses showed delayed activity.

### 2.8. CalcuSyn Assay for Combination Therapy Assay

The CalcuSyn method was previously validated in our laboratory for antimalarial drug interaction analysis [29,30]. The atovaquone–proguanil combination was used to establish the robustness of the CalcuSyn drug interactivity software. The compounds were combined in accordance to the previously determined IC_50_ values. Briefly, a two-fold serial dilution was carried out based on IC_50_ values. The mid-point equated to the previously determined IC_50_ values for each drug. The 1% ring stage parasites were treated with either atovaquone or proguanil solely or in combination. The ratio for the combination was 1:3500 (atovaquone–proguanil) (Table 4) (Figure S7) [29,30]. 

The samples were analysed after 72 h using the SYBR Green plate reader method and further analysed with CalcuSyn Biosoft version 2.1 software. The dose–effect curve, median effect plot, and isobologram (see Figure S7 for data) showed improved potency for the combination of atovaquone and proguanil in comparison to the individual drugs for the validation of the methodology. The combination index (CI) at IC_50_, IC_75_, and IC_90_ was used to determine synergism where CI > 1 was classified as antagonism, CI = 1 additive, and CI < 1 synergism [31]. CI values of 0.215, 0.388, and 0.698 at IC_50_, IC_75_, and IC_90_, respectively, proved the strong synergistic interaction between atovaquone and proguanil.

Upon validation of the atovaquone–proguanil combination, the interactions between the lead compound **2** and artemisinin (12.5:1), artemether (2:1), or doxycycline (1:300) were studied. The doses were selected based on the respective predetermined IC_50_ values (from 72 h assay) of compound **2**. The combination of compound **2** and artemisinin was classified as an antagonist at IC_90_ and IC_75_, while a slight synergism was observed at IC_50_. The combination of compound **2** and doxycycline was classified as nearly additive at IC_90_ and observed to be antagonist at IC_50_ and IC_75_. Synergism was observed between compound **2** and artemether at IC_90_ and nearly additive at IC_50_ and IC_75_ (Table 5) and dose–response data (Figure S8).

## 3. Discussion

The 4-aminoquinoline hydrazone compounds were initially developed as anticancer drugs [23]. In this study, these compounds were shown to have antimalarial activity with IC_50_ values ranging from 0.60 to 49 μM against the multidrug-resistant K1 strain of *P. falciparum* after 48 h of drug exposure. The time-course experiment indicated that the activity of the compounds became more pronounced as time progressed. We then assessed the activity of the lead compounds (chosen based on their activity at 48 h assay and structural differences) at 72 h. We observed that the compounds were now active at low nano-molar IC_50_ values with the most active compounds **1** and **2** having IC_50_ values of 25 nM and 32 nM, respectively. This suggested that the compounds were potent but were either acting more effectively with some delay or were stage-specific. The latter was indeed confirmed, as most of our compounds showed predominant sensitivity for the ring stage.

One of the key fundamentals when developing antimalaria treatments is that the drug candidate must be well-tolerated and safe for use, especially for pregnant women and children. We investigated the toxicity of the 4-aminoquinoline hydrazones using the in vitro MTT assay in HepG2 and MDBK cell lines. The compounds displayed toxicity with low IC_50_ values ranging from 0.87 to 11.1 μM for HepG2 and 1.66 to 11.7 μM for MDBK. Interestingly, when the incubation time was increased on the *P. falciparum* K1 strain dose–response assay to 72 h, it was observed that all of the compounds except for compound **4** and **13** (Figure S7) were safe, with therapeutic selectivity indices of >30 for the MDBK cell line. Although the assessed toxicity effect suggested that the compounds might be safe, an appropriate toxicity investigation to support these results can only be concluded with the use of in vivo models. The lead compound **2** was evaluated with the *P. yoelii* NL mouse model and shown to be efficacious, with a daily dose of 20 mg/kg clearing the parasitaemia by day 1 post-treatment. The mice were fully cured from malaria infection, indicating that the 20 mg/kg dose is still higher than the minimum effective concentration; therefore, future studies should include a lower dose range (1–10 mg/kg) to identify the minimum in vivo effective concentration.

The use of combination regimes has been shown to have a therapeutic advantage in comparison to monotherapy. The World Health Organisation (WHO) recommends the use of artemisinin-based combinations to address the failures of monotherapy. The combination regimes can kill the majority of parasites over several days by one mechanism and the partner drug can prevent recrudescence. Three existing antimalarial drugs, artemether, artemisinin, and doxycycline, were investigated for their interaction with compound **2**. Compound **2** was found to exhibit mild synergistic activity (additive) with doxycycline. The synergistic activity of compound **2** with artemether presents an exciting option for antimalarial therapy as the WHO recommends artemisinin and derivatives-based combinations to increase antimalarial potency and prolong the shelf-lives of the frontline antimalarials.

## 4. Materials and Methods

### 4.1. Drug Preparation and Synthesis

Compounds **1**–**16** were synthesised using the experimental procedures described previously [23]. The purity of the compounds was checked by ^1^H and ^13^C NMR spectroscopy and LC-MS and were found to be >95% pure prior to biological experiments. NMR spectra were recorded on a Bruker 300 MHz spectrometer. LC-MS was carried out using the Acquity UPLC H-class system. The mass spectrometry data were acquired in the positive (ES^+^) and negative (ES^−^) modes scanned from 100 to 1000 *m*/*z*. The LC data were obtained for Waters Acquity UPLC PDA detector scanning from 210 to 400 nm. Drug stock solutions were prepared in DMSO at 50 mM. The stock solutions were stored at 4 °C. Serial dilution in a complete medium was prepared from stock solutions immediately before use.

#### 4.1.1. *Plasmodium falciparum* Parasite Cultivation

The K1, 3D7 (from MR4 and in-lab maintained and isolated clone A10), and Dd2 strain of *P. falciparum* were cultured with RPMI 1640 medium containing 25 mM HEPES and 0.3 g/L l-glutamine (Gibco, Life Technologies, Paisley, UK) [32,33]. The medium was supplemented with 2.5 g AlbuMax (Sigma, Gillingham, UK) and sterile solutions of 2.5 mL hypoxanthine (Sigma, UK), 2.5 mL 40% glucose (Dextrose Anhydrous, Fisher Scientific, Loughborough, UK), and 0.5 mL gentamycin (Sigma, UK). Human erythrocytes served as host cells. Cultures were maintained at 37 °C under gas mixture of 5% CO_2_, 5% O_2_ in N_2_ (BOC Limited, Manchester, UK).

#### 4.1.2. Synchronization of K1 *P. falciparum*

K1 strain of *P. falciparum* maintained at 5% haematocrit was used to establish synchrony. The culture was cultivated to ~8% parasitaemia, with the prevalence of ring stages. The culture was centrifuged at 3500 rpm for 5 min. The supernatant was removed and parasitized RBCs were re-suspended in 5% sorbitol, incubated at room temperature for 5 min, and then centrifuged at 3500 rpm for 5 min. The supernatant was removed and the pellet was washed 3 times with complete medium before setting up a new culture.

#### 4.1.3. Determination of IC_50_ in *P. falciparum*

In total, 1% of infected RBCs at the ring or trophozoites stage was exposed to nine dilutions (four-fold serial dilution) of compounds from 50 mM stock solutions in DMSO. A total of 100 µL of culture volume was added in 96-well plates with 100 µL drug dilutions. Plates were either incubated for 48 h or 72 h at 37 °C, 5% CO_2_, 5% O_2_ in N_2_. Following the incubation, 150 µL of the medium was carefully removed and 150 µL of the 5× SYBR Green solution (prepared by adding 2 µL of 10,000× SYBR Green in 4 mL of PBS) was added to each well. Plates were kept in the dark for 45 min. Fluorescence intensity was measured using a microplate reader (Genius Tecan) with an excitation of 485 nm and an emission of 535 nm.

#### 4.1.4. Time-Course Assay

The time-course assay was used to define the activity timelines of the 4-aminoquinoline hydrazone compounds. Parasite growth of K1 strain *P. falciparum* in the presence of 4-aminoquinoline hydrazone compounds was assessed on asynchronous culture. The growth impairment of K1 culture was initiated at 1% parasitaemia for 24, 48, and 72 h. The assay was analysed by flow cytometry as described below.

#### 4.1.5. Flow Cytometry

Following drug efficacy experiments, 100 µL from the control and drug-treated samples on a 96-well plate were transferred to a micro-centrifuge tube containing 1 mL of PBS. The samples were centrifuged at 1200 rpm for 2 min. The supernatant was removed and the samples were re-suspended in 1 mL of 5X SYBR Green solution and incubated in the dark for 20 min at room temperature. After staining, the samples were centrifuged for 2 min at 1200 rpm and the supernatant was discarded. The samples were fixed with 0.4% formaldehyde solution (250 µL). The samples were placed in the fridge and incubated at 4 °C for 10–15 min and subsequently washed three times with PBS. The pellet was re-suspended in PBS (1 mL) and parasitaemia was determined by SYBR Green fluorescence using the FITC channel of the BD FACs Verse flow cytometer system (Blue laser, excitation laser line 488 nM Exmax 494/EMmax 520 nM) and cell size (forward scatter, FSC-A). Fluorescent events in drug-treated samples were compared with infected and uninfected blood counterparts and gated accordingly to obtain the percentage parasitaemia.

#### 4.1.6. MTT Assay for Testing Cell Cytotoxicity

Human hepatoma (HepG2) and the Madin–Darby bovine kidney (MDBK) cell lines were purchased from American Type Culture Collection (ATCC), USA. The MDBK cell line was cultured with Dulbecco’s Modified Eagle’s Medium (DMEM) containing 4.5 g/L (+)-D-glucose and L-glutamine, while the HepG2 cell line was cultured with RPMI 1640 containing 2 mM L-glutamine and HEPES. The culture medium was supplemented with 10% Fetal Bovine Serum (FBS) and 1% penicillin. Cells were maintained in the presence of 5% CO_2_ atmosphere at 37 °C. The assay was carried out on a 96-well plate, with each well containing 4 × 10^3^ cells. Plates were firstly incubated for 24 h at 37 °C to ensure cell adherence. The cells were then treated with 100 µL of the drug solution prepared at various concentrations and further incubated for 5 days. After the incubation period, 50 µL of the MTT (3-(4,5-dimethylthiazol-2-yl)-2,5-diphenyltetrazolium bromide, Sigma, UK) solution was added to each well and incubated for a further 3 h. The MTT solution and medium were aspirated, 150 µL of DMSO was added to each well, and the results were read on the Ascent plate reader.

#### 4.1.7. Stage Specificity Assay

*P. falciparum* K1 was synchronised twice with 5% sorbitol at 0 h and 31 h to obtain early rings (up to 3 h old) [34]. In order to obtain late trophozoites to early schizonts, the culture was synchronised twice at 0 h and 6 h. Each synchronous stage was diluted to 1% parasitaemia. On a 96-well plate, the two synchronous stages were incubated for 24 h at 37 °C with a two-fold serial dilution of the six most active 4-aminoquinoline hydrazone compounds. The dilution ranged from 1.6 to 100× fold of the previously determined IC_50_ of each compound in a standard 48 h assay. After a 24 h incubation period, the plates were washed 4 times resulting in about a 100-fold dilution of the free compound. The plates were further incubated for an additional 24 h at 37 °C and analysed using SYBR Green staining assays as previously described [35].

#### 4.1.8. Derivation of the Dose–Response Curves and IC_50_ Values

IC_50_ values were determined using Graph Pad Prism 5.0. Values were calculated using non-linear regression by using log-transformed drug concentration plotted against dose–response. Parasitaemia was calculated and normalised relative to the response on the controls (cultures without drug). 

#### 4.1.9. Drug Interaction Assay for 4-Aminoquinoline Hydrazones

The drug combination was determined using the CalcuSyn method. This method relies on a predetermined IC_50_, which was conducted as previously explained. In total, 0.12–8 times the ED_50_ of each drug was combined together and 2-fold serial dilution was carried out. Ring stage parasites were treated with drug dilution and incubated for 72 h in a 96-well plate format. Drug susceptibility was determined by the SYBR Green plate reader method, while the median effect was determined using the CalcuSyn method. The drug interaction was determined according to the median effect principle by Chou Ting-Chao [31,36]. The CalcuSyn software is able to generate the isobologram plots, dose–effect curves, median effect curves, and combination indexes (CI) to assess eventual drug to drug interaction. The combination index, which represents the pharmacological interaction, taking into account both the potency and the shape of the dose–response curve, has been extensively studied and can be interpreted according to CI < 1 (synergy), CI = 1 (additive), and CI > 1 (antagonist) [31]. 

#### 4.1.10. *P. yoelii* NL Mice Screening with Compound **2**

C57BL/6 mice were purchased from Charles River, UK, and were maintained at the University of Manchester in individually ventilated cages. A total of six mice were used for this experiment and kept in the cage (two in each cage) with randomisation of mice given antimalarial drugs and vehicle control. The mice were maintained in individually ventilated cages with food and water ad libitum. Male and females were kept within separate cages during the course of the experiment. The mice generally weighed 18–20 g at the start of the experiment. Cryopreserved *P. yoelii* NL parasites were passed once through mice before the infection experiment. Male/female mice 6–10 weeks in age were infected with 1 × 10^4^ parasitised red blood cells (pRBCs) via intravenous injection in the tail vein. Parasitaemia growth was monitored via Giemsa-stained blood smears. Three groups of mice (placebo, drug dose of 5 mg/kg or 20 mg/kg) were used for this experiment. Compound **2** was injected once a day for 4 days starting at the day the infection level reached ~5% parasitaemia. After the injection, mice were monitored for 20 days to detect eventual signs of both infection rebound and toxicity. 

All animal work was approved following local ethical review by the Manchester Animal Procedures and ethics committee and was performed in strict accordance with the United Kingdom Home Office Animals (Scientific Procedure) Act 1986 (License No. P8829D3B4).

## 5. Conclusions

The 4-aminoquinoline hydrazone compounds showed good activity and low cytotoxicity, suggesting that they can be used as lead compounds as novel antimalarials. Moreover, they showed a rapid onset of action, which is very important for relieving patient symptoms early and minimising parasite resistance [37,38]. The activity of these compounds warrants their further investigation as potential drugs in chemotherapy. The WHO recommends ACT to confront drug-resistant *P. falciparum* malaria [1]. Moreover, ACT can improve antimalarial efficacy, favourable synergistic interaction, and decreased toxicity [39,40]. Therefore, it is of interest to further evaluate the 4-aminoquinoline hydrazone compounds as partner drugs for combination therapy.

## Figures and Tables

**Table 1:** Inhibition of *P. falciparum* asexual growth by the 4-aminoquinoline hydrazones (**1**–**16**) was measured after an incubation period of 48 h using the SYBR Green-based plate reader assay to give IC_50_ values (µM). Compound toxicity was measured in HepG2 and MDBK cells using the MTT assay. Both experiments were performed in triplicate to give standard error bars. Data were analysed using Graph Pad Prism. For some weak active compounds, the replicates showed poor reproducibility (wide) and the SE could not be calculated.

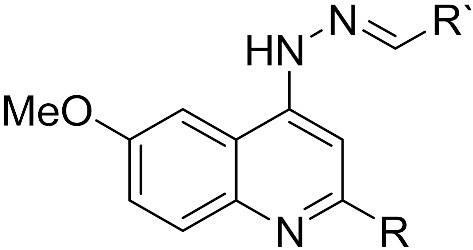					
			*Plasmodium* IC_50_(µM) ± SE	Cell Toxicity IC_50_(µM) ± SE	Cell Toxicity IC_50_(µM) ± SE
Compound	R	R’		HepG2	MDBK
**1**	-Me	4-Fluorophenyl	0.612 ± 0.35	0.872 ± 0.79	3.77 ± 1.5
**2**	-Me	Phenyl	2.26 ± 2.2	1.90 ± 0.11	3.44 ± 2.7
**3**	-Me	3-Pyridinyl	3.29 ± 1.6	3.00 ± 0.27	7.34 ± 7.6
**4**	-Me	2-Nitrofuranyl	0.600 ± 0.84	1.59 ± 0.18	1.66 ± 0.21
**5**	-Me	2-Hydroxy-3-methoxyphenyl	3.82 (wide)	6.73 ± 0.73	>25
**6**	-Me	4-Imidazoyl	48.8 (wide)	7.26 ± 1.00	11.7 ± 2.5
**7**	-Me	4-Nitrophenyl	20.1 (wide)	1.69 (wide)	6.68 ± 1.7
**8**	-Me	4-Hydroxyphenyl	22.3 (wide)	1.97 ± 0.42	3.73 ± 1.2
**9**	-Me	4-Benzoic acid	>200	>25	>25
**10**	-Me	3-Hydroxyphenyl	18.9 (wide)	2.64 ± 0.20	9.71 ± 3.5
**11**	-Me	3,5-Dihydroxylphenyl	35.9 ± 43	11.1 ± 2.9	>25
**12**	-Ph	2-Hydroxy-3-methoxyphenyl	5.67 ± 13	1.80 ± 0.78	6.60
**13**	-Ph	Benzyl	3.33 ± 2.4	>25	>25
**14**	-Ph	4-Nitrophenyl	2.20 ± 1.5	0.89 ± 0.13	4.24 ± 1.0
**15**	-Ph	4-Imidazoyl	28.3 ± 29	2.21 ± 0.15	9.31 ± 3.5
**16**	-Ph	4-*N*,*N*-dimethylaniline	8.06 ± 4.5	4.15 ± 2.4	6.24 (wide)
Chloroquine			0.199 ± 0.04		
Cisplatin				3.92 ± 0.64	9.15 ± 3.5

**Table 2:** Inhibition of *P. falciparum* asexual growth in 72 h. IC_50_ values (µM) of 4-aminoquinoline hydrazone compounds measured after an incubation period of 72 h using SYBR Green-based plate reader assay. Experiments were performed three times, giving the standard error (SE). The selectivity indices calculated from cell viability IC_50_ determined in Table 1, which was carried out for five days. Data were analysed using Graph Pad Prism. (Wide: standard error could not be calculated). The selectivity index of compounds **5** and **13** is calculated by assuming cytotoxicity IC_50_ = 25 μM (the highest concentration used for the experiment).

Compound	*Plasmodium* IC_50_ (µM) ± SE	Selectivity Indices (MDBK)	Selectivity Indices (HepG2)
**1**	0.0257 (wide)	147	34
**2**	0.0329 ± 0.007	101	58
**3**	0.175 ± 0.04	42	17
**4**	0.219 ± 0.02	7	7
**5**	0.174 ± 0.03	>143	39
**12**	0.133 ± 0.06	50	14
**13**	1.61 (wide)	>15	>15
**14**	0.176 ± 0.06	31	5

**Table 3:** The effective dose of compounds **1** and **2** tested in 4-fold serial dilutions from 0.00305 to 200 µM on *P. falciparum* 3D7 and Dd2 strains. The parasites were treated with the compounds for 72 h. The growth was read using the SYBR Green-based plate reader assay. The experiment was compared to the K1 assay (also in Table 2). The experiments were performed at each concentration three times in triplicate. Data were analysed with GraphPad prism. (Wide: standard error could not be calculated).

Compounds	3D7 IC_50_ ± SE (µM)	Dd2 IC_50_ ± SE (µM)	K1 IC_50_ ± SE (µM)
**1**	0.183 ± 1.04	0.133 ± 1.18	0.0257 (wide)
**2**	0.0554 (wide)	0.0244 (wide)	0.0329 ± 0.007

**Table 4:** CalcuSyn outputs for the atovaquone–proguanil combination. The combinatory index values (CI) are shown for atovaquone and proguanil at the IC_50_, IC_75_, and IC_90_ levels of inhibition. The m and r values are also reported for all sets of data. The m value refers to the kinetic order and shape of the curve: m = 1, >1, and <1 indicate hyperbolic, sigmoidal, and negative sigmoidal shape, respectively. The r value represents the linear correlation coefficient for the median effect plot and indicates conformity to the mass action law. (N/A-not measured).

Drug Combination	CI Values at				
	IC_50_	IC_75_	IC_90_	m	r
**Atovaquone**	N/A	N/A	N/A	0.652	0.917
**Proguanil**	N/A	N/A	N/A	0.565	0.983
**Combination**	0.215	0.388	0.698	0.483	0.880

**Table 5:** CalcuSyn outputs for the combination of known antimalarial drugs and compound **2**. The combinatory index values (CI) are shown at the IC_50_, IC_75_, and IC_90_ levels of inhibition. The m and r values are also reported for all sets of data. The m values refer to the kinetic order and shape of the curve: m = 1, >1, and <1 indicate hyperbolic, sigmoidal, and negative sigmoidal shape, respectively. The r value represents the linear correlation coefficient for the median effect plot and indicates conformity to the mass action law.

Drug Combination	CI Values at				
	IC_50_	IC_75_	IC_90_	m	r
**2 + Artemisinin**	0.822	1.907	4.563	0.263	0.869
**2 + Artemether**	2.484	1.148	0.599	0.698	0.883
**2 + Doxycycline**	1.285	1.112	0.963	0.467	0.973

## Data Availability

The data presented in this study are available in the main text or the Supplementary Materials.

## Supplementary Materials

The following supporting information can be downloaded at: https://www.mdpi.com/article/10.3390/molecules28186471/s1, Figure S1: Structures of the 4-aminoquinoline hydrazone compounds that were tested for their antimalarial activities; Figure S2: The effect of the 4-aminoquinoline hydrazone compounds along with chloroquine tested on the *P. falciparum* K1 strain (trophozoite stage). The 4-aminoquinoline hydrazones were tested in 4-fold dilution of dose range from 0.00305 to 200 µM, while chloroquine was tested in a dose of 9.3–600 nM. The compounds were incubated for 48 h and read using SYBER Green-based plate reader assay. The experiments were performed three times in triplicates. The data were analysed using GraphPad prism, Figure S3: The effect dose of the 4-aminoquinoline hydrazone compounds tested on the *P. falciparum* K1 strain (ring stage). The compounds were tested in 4-fold dilution of dose range from 0.00305 to 200 µM, incubated for 72 h and read using SYBER Green-based plate reader assay. The experiments were performed three times in triplicate. The data were analysed using GraphPad prism; Figure S4: MTT assay was assessed on Hep G2 cells. The cells were seeded at 4000 cells per well. The HepG2 cells were incubated with 2-fold serial dilution of the compounds at dose range of 0.39–25 µM for 5 days. Cell viability was determined using the standard MTT assay. Data were analysed using GraphPad prism. The experiments were performed three times; each concentration was repeated three times per experiment; Figure S5: MTT assay was assessed on MDBK cells. The cells were seeded at 4000 cells per well. The MDBK cells were incubated with 2-fold serial dilution of compounds at dose range of 0.39–25 µM for 5 days. Cell viability was determined using the standard MTT assay. Data were analysed using GraphPad prism. The experiments were performed three times; each concentration was repeated three times per experiment; Figure S6: The effect dose of the lead 4-aminoquinoline hydrazone compounds **1** and **2** tested on the *P. falciparum* 3D7 ig A10 (A) and Dd2 (B) in 4-fold dilution of dose range from 0.00305 to 200 µM. The compounds were incubated for 72 h and read using SYBER Green-based plate reader assay. The experiments were performed three times in triplicate. The data were analysed using GraphPad prism; Figure S7: Dose–effect analysis of atovaquone and proguanil combination on *P. falciparum* K1 strain. The dose–response data were analysed with CalcuSyn software to give a dose–effect curve and isobologram graph; Figure S8: Dose–effect analysis of compound **2** and artemisinin, artemether and doxycycline combination on *P. falciparum* K1 strain. The dose–response data were analysed with CalcuSyn software to give dose–effect curve and isobologram graph.
