# Supplementary material for: Evaluation of 4-Aminoquinoline Hydrazone Analogues as Potential Leads for Drug-Resistant Malaria
Source: Molecules. 2023 Sep 6;28(18):6471. doi: 10.3390/molecules28186471 (PMC10534891; doi:10.3390/molecules28186471)
Supplement: Supplementary file 1 [file molecules-28-06471-s001.zip › molecules-2553874-supplementary.pdf]

Supplementary Materials

Evaluation of 4-Aminoquinoline Hydrazone Analogues as Potential Leads for Drug-Resistant Malaria

Rachael N. Magwaza [1,2], Muna Abubaker [2], Buthaina Hussain [3], Michael Haley [4], Kevin Couper [4], Sally Freeman [1,*] and Niroshini J. Nirmalan [2,*]

[1] Division of Pharmacy and Optometry, School of Health Sciences, University of Manchester, Manchester M13 9PT, UK
[2] School of Science, Engineering and Environment, University of Salford, Manchester M5 4WT, UK; r.n.magwaza@salford.ac.uk (R.N.M.); m.abubaker2@salford.ac.uk (M.A.)
[3] Faculty of Pharmacy, Al-Zaytoonah University of Jordan, Amman 17138, Jordan; buthina.hussein@zuj.edu.jo
[4] School of Biological Sciences, Lydia Becker Institute of Immunology and Infection, University of Manchester, Manchester M13 9PT, UK; michael.haley@manchester.ac.uk (M.H.); kevin.couper@manchester.ac.uk (K.C.)
* Correspondence: sally.freeman@manchester.ac.uk (S.F.); n.j.nirmalan@salford.ac.uk (N.J.N.)

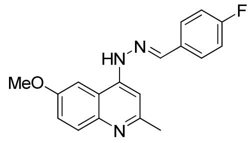

1

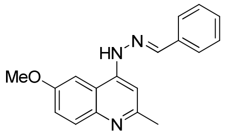

2

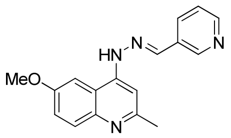

3

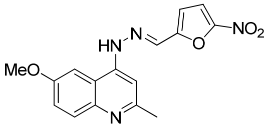

4

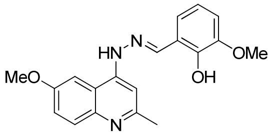

5

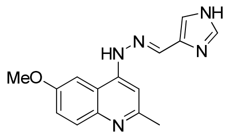

6

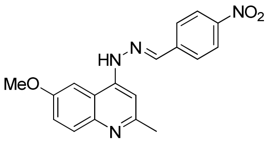

7

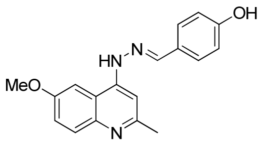

8

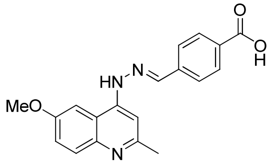

9

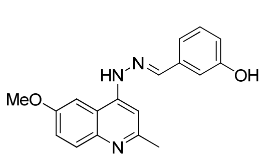

10

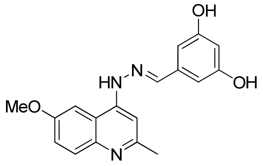

11

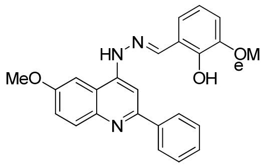

12

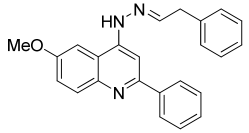

13

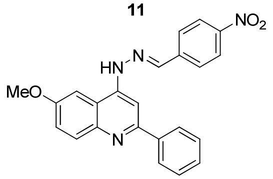

14

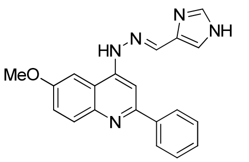

15

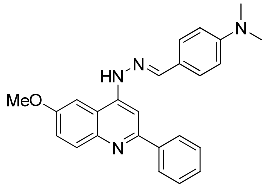

16

Figure S1. Structures of the 4-aminoquinoline hydrazone compounds that were tested for their antimalarial activities.

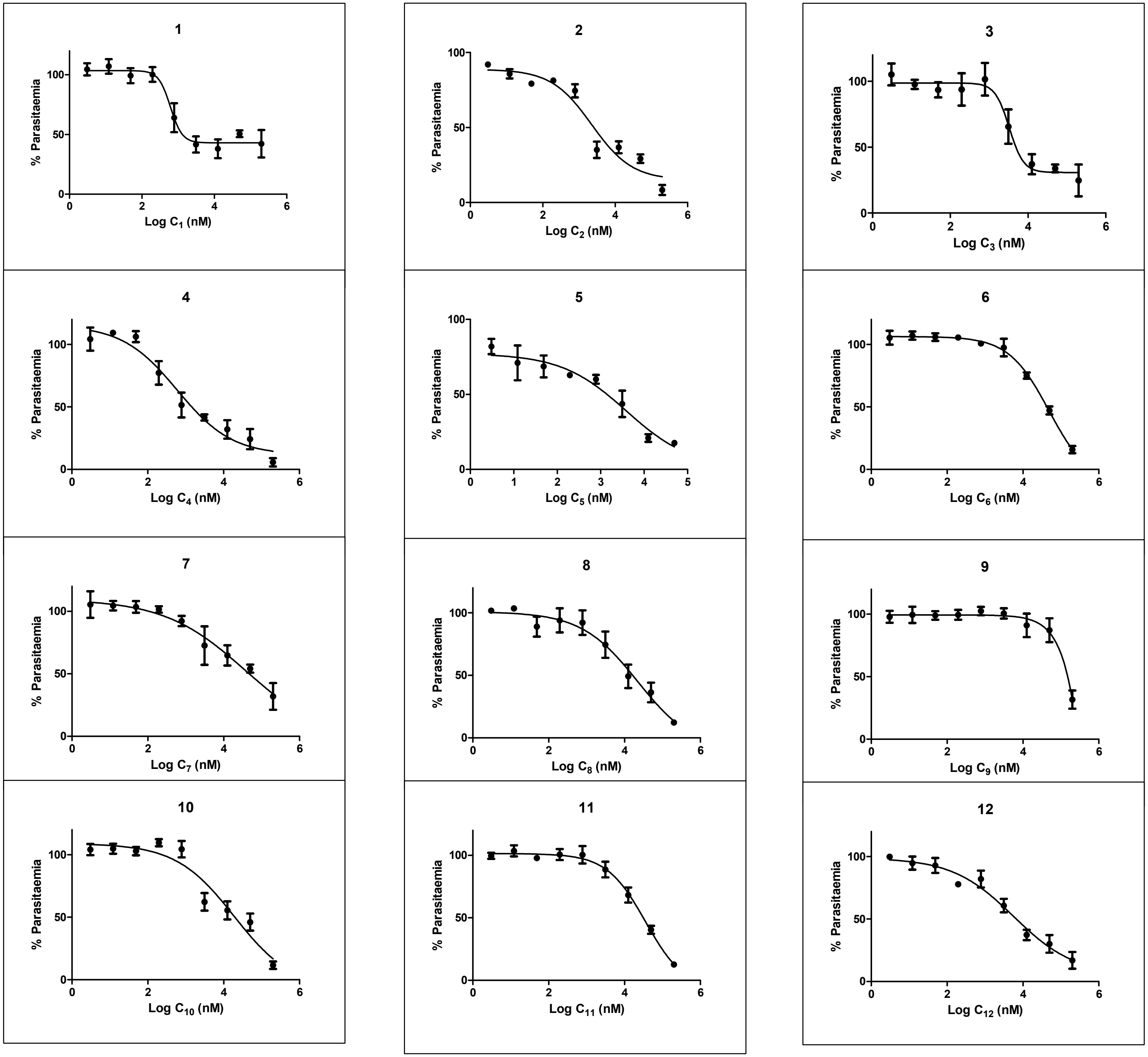

1
% Parasitaemia
Log C1 (nM)
2
% Parasitaemia
Log C2 (nM)
3
% Parasitaemia
Log C3 (nM)
4
% Parasitaemia
Log C4 (nM)
5
% Parasitaemia
Log C5 (nM)
6
% Parasitaemia
Log C6 (nM)
7
% Parasitaemia
Log C7 (nM)
8
% Parasitaemia
Log C8 (nM)
9
% Parasitaemia
Log C9 (nM)
10
% Parasitaemia
Log C10 (nM)
11
% Parasitaemia
Log C11 (nM)
12
% Parasitaemia
Log C12 (nM)

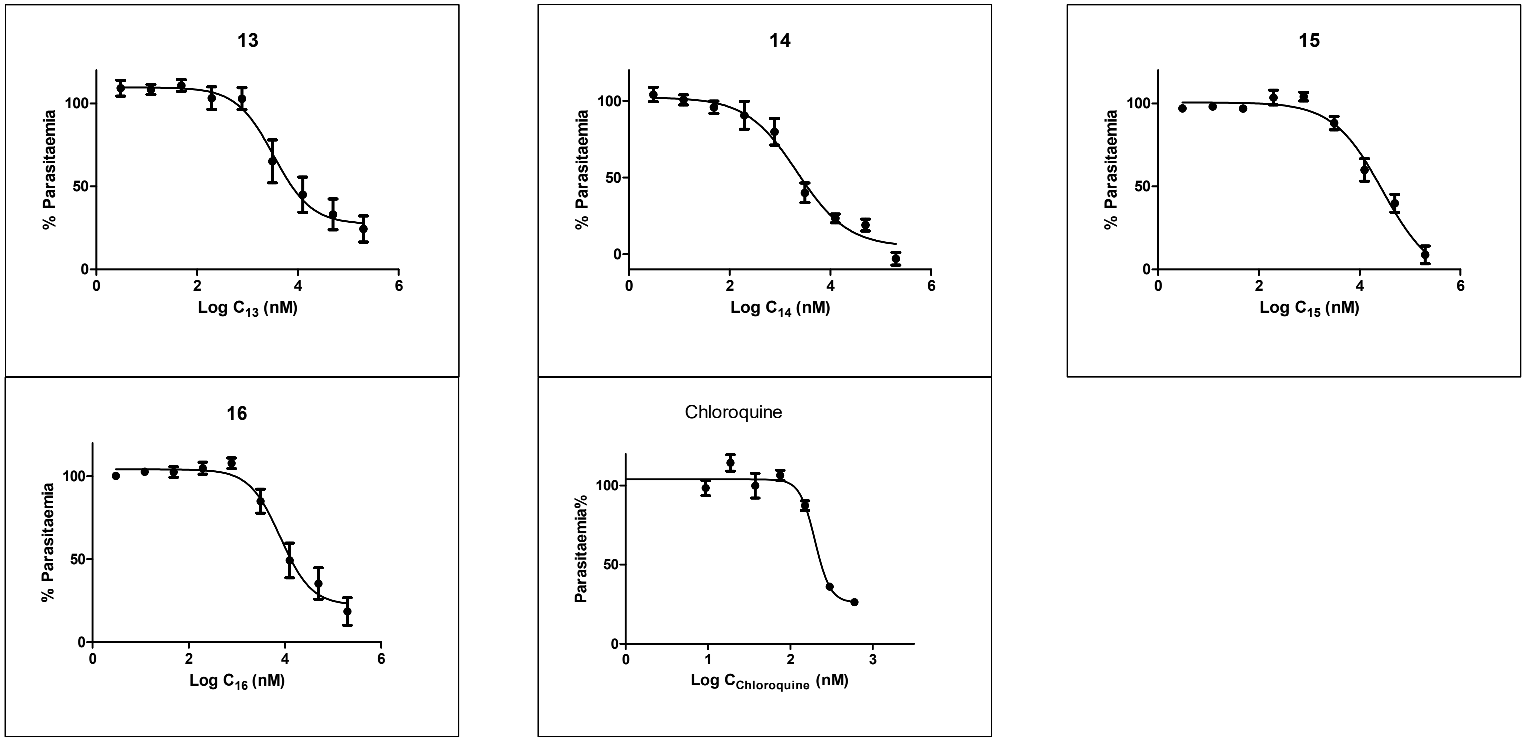

Figure S2. The effect of the 4-aminoquinoline hydrazone compounds along with chloroquine tested on the *P. falciparum* K1 strain (trophozoite stage). The 4-aminoquinoline hydrazones were tested in four-fold serial dilutions from 0.00305 - 200 µM, while Chloroquine was tested at a dose of 9.3 - 600 nM. The compounds were incubated for 48 hours and read using SYBER green-based plate reader assay. The experiments were performed three times in triplicates. The data was analysed using GraphPad prism.

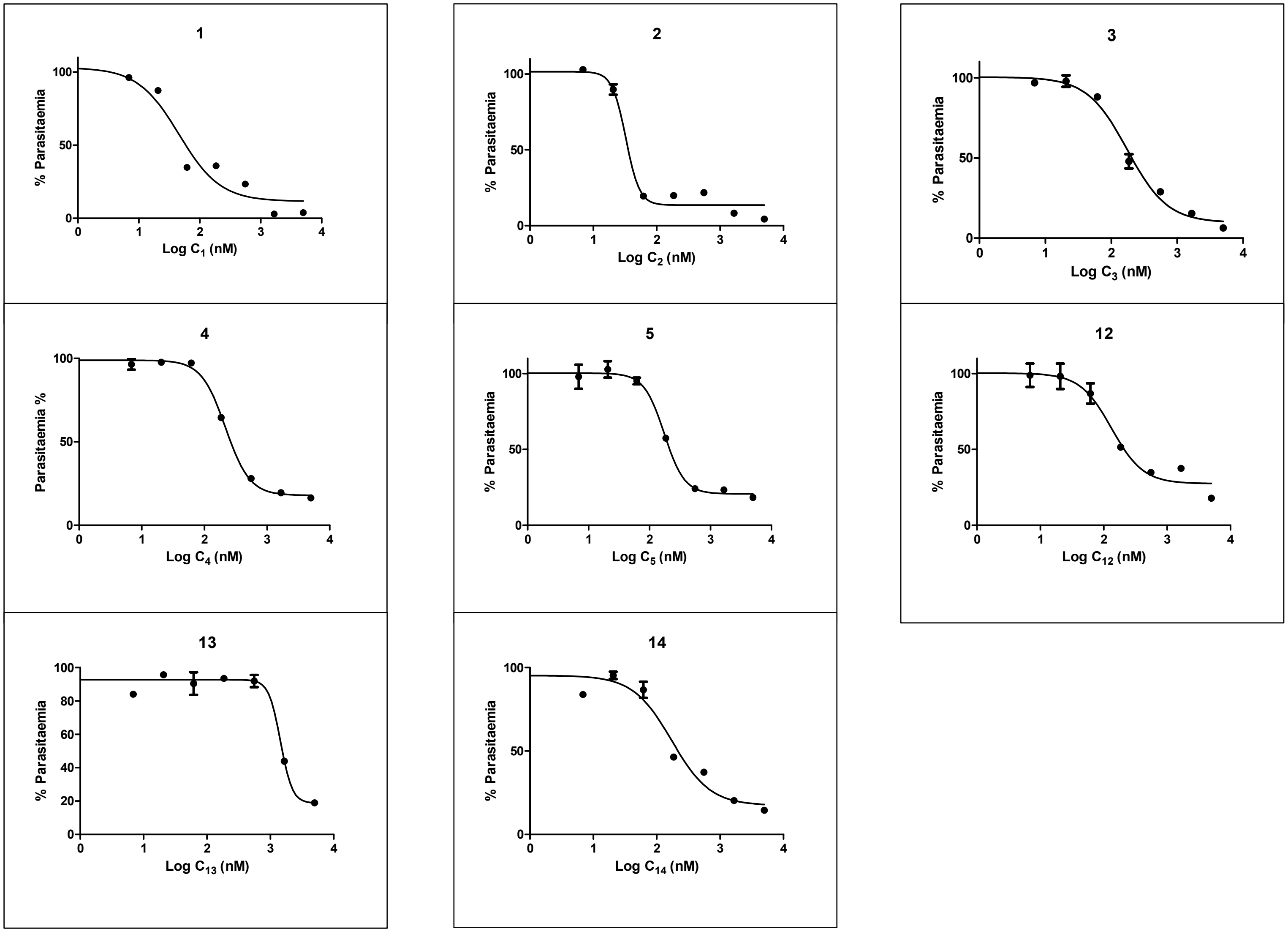

Figure S3. The effect dose of the 4-aminoquinoline hydrazone compounds tested on the *P. falciparum* K1 strain (ring stage). The compounds were tested in four-fold serial dilutions from 0.00305 - 200 μM, incubated for 72 hours and read using SYBER green-based plate reader assay. The experiments were performed three times in triplicates. The data was analysed using GraphPad prism.

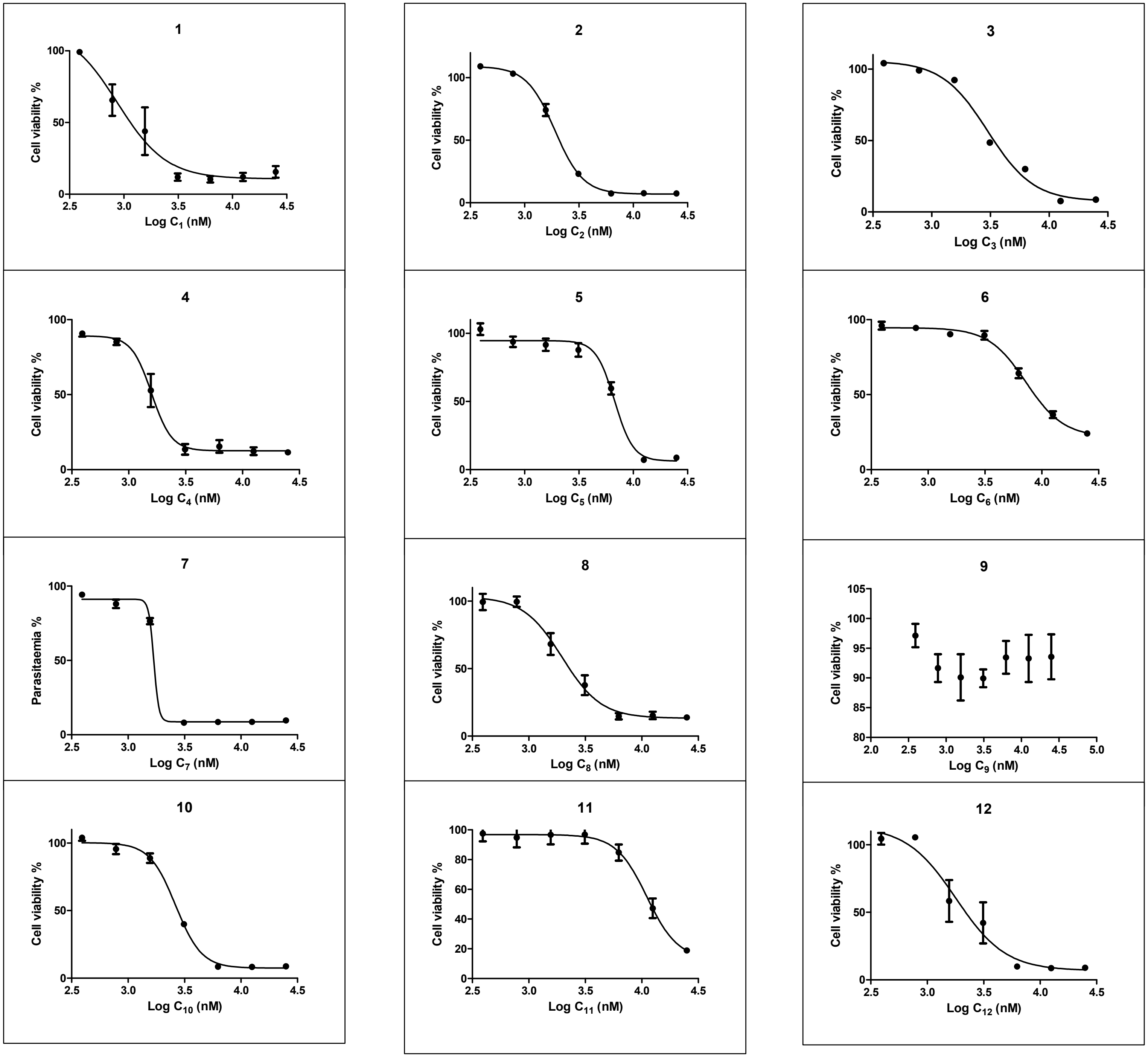

1
Cell viability %
Log C1 (nM)
2
Cell viability %
Log C2 (nM)
3
Cell viability %
Log C3 (nM)
4
Cell viability %
Log C4 (nM)
5
Cell viability %
Log C5 (nM)
6
Cell viability %
Log C6 (nM)
7
Parasitaemia %
Log C7 (nM)
8
Cell viability %
Log C8 (nM)
9
Cell viability %
Log C9 (nM)
10
Cell viability %
Log C10 (nM)
11
Cell viability %
Log C11 (nM)
12
Cell viability %
Log C12 (nM)

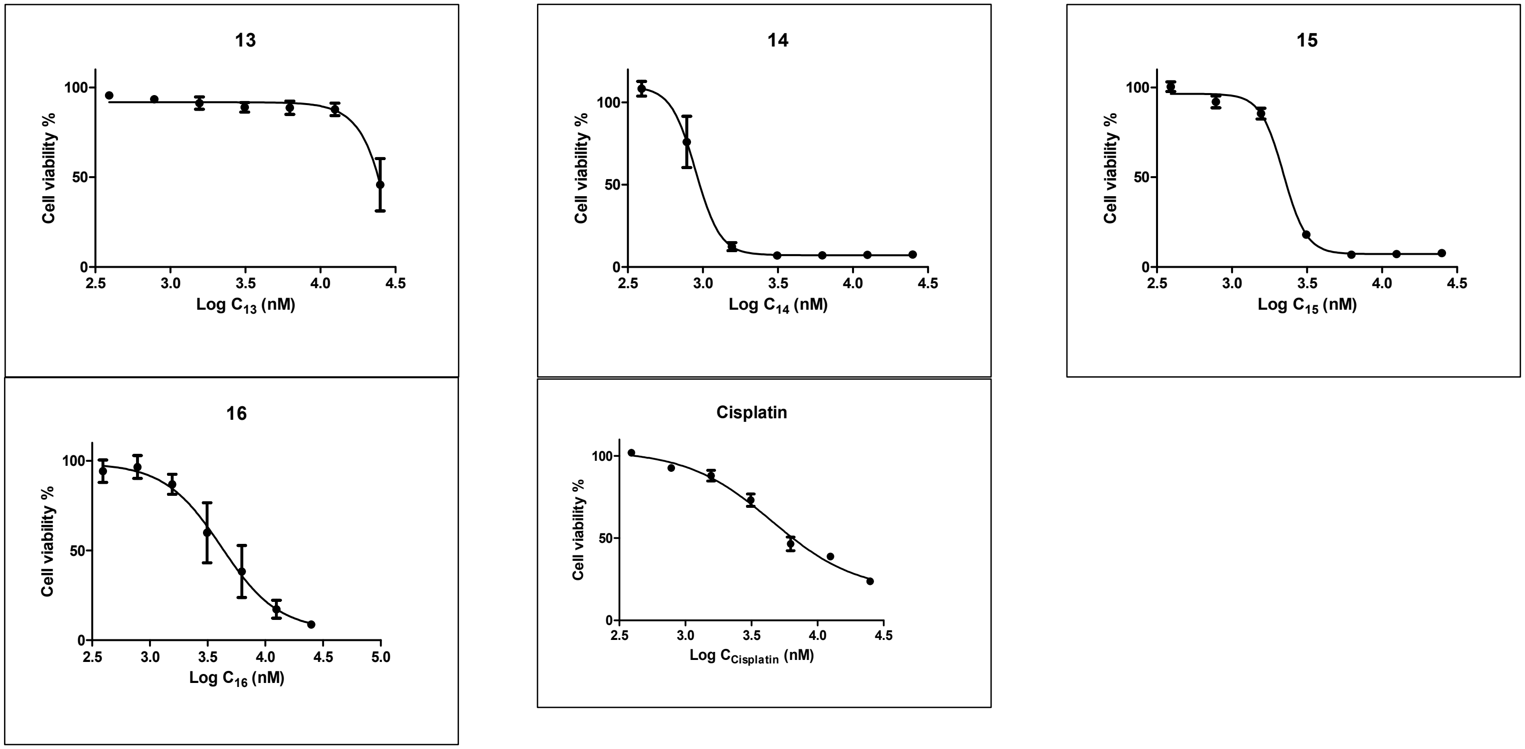

Figure S4. MTT assay was assessed on Hep G2 cells. The cells were seeded at 4000 cells per well. The HepG2 cells were incubated with two-fold serial dilution of the compounds at dose range of 0.39 -25 μM for 5 days. Cell viability was determined using the standard MTT assay. Data was analysed using GraphPad prism. The experiments were performed three times; each concentration was repeated three times per experiment.

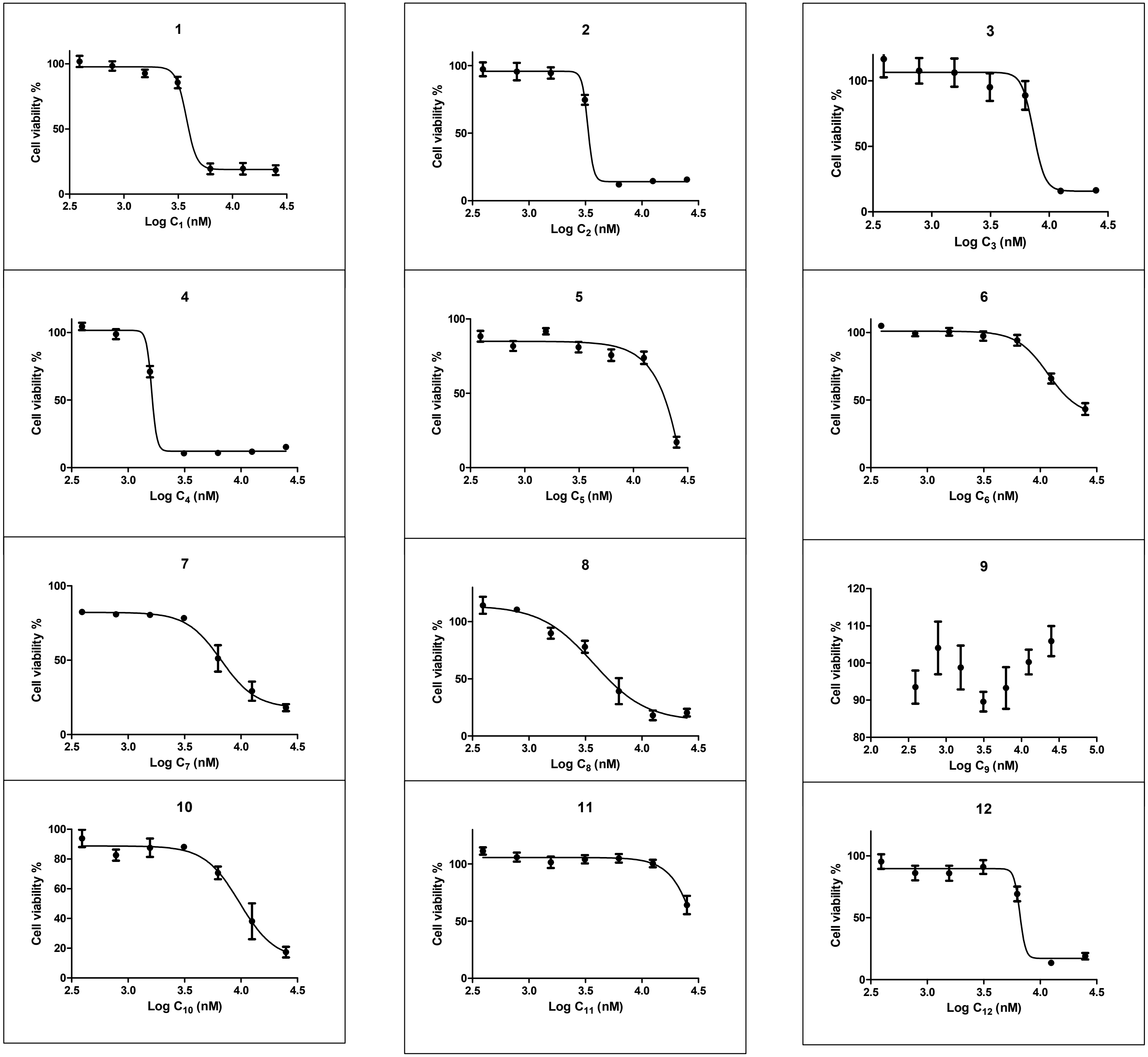

1
Cell viability %
0
50
100
2.5 3.0 3.5 4.0 4.5
Log C1 (nM)
2
Cell viability %
0
50
100
2.5 3.0 3.5 4.0 4.5
Log C2 (nM)
3
Cell viability %
0
50
100
2.5 3.0 3.5 4.0 4.5
Log C3 (nM)
4
Cell viability %
0
50
100
2.5 3.0 3.5 4.0 4.5
Log C4 (nM)
5
Cell viability %
0
50
100
2.5 3.0 3.5 4.0 4.5
Log C5 (nM)
6
Cell viability %
0
50
100
2.5 3.0 3.5 4.0 4.5
Log C6 (nM)
7
Cell viability %
0
50
100
2.5 3.0 3.5 4.0 4.5
Log C7 (nM)
8
Cell viability %
0
50
100
2.5 3.0 3.5 4.0 4.5
Log C8 (nM)
9
Cell viability %
80
90
100
110
120
2.0 2.5 3.0 3.5 4.0 4.5 5.0
Log C9 (nM)
10
Cell viability %
0
20
40
60
80
100
2.5 3.0 3.5 4.0 4.5
Log C10 (nM)
11
Cell viability %
0
50
100
2.5 3.0 3.5 4.0 4.5
Log C11 (nM)
12
Cell viability %
0
50
100
2.5 3.0 3.5 4.0 4.5
Log C12 (nM)

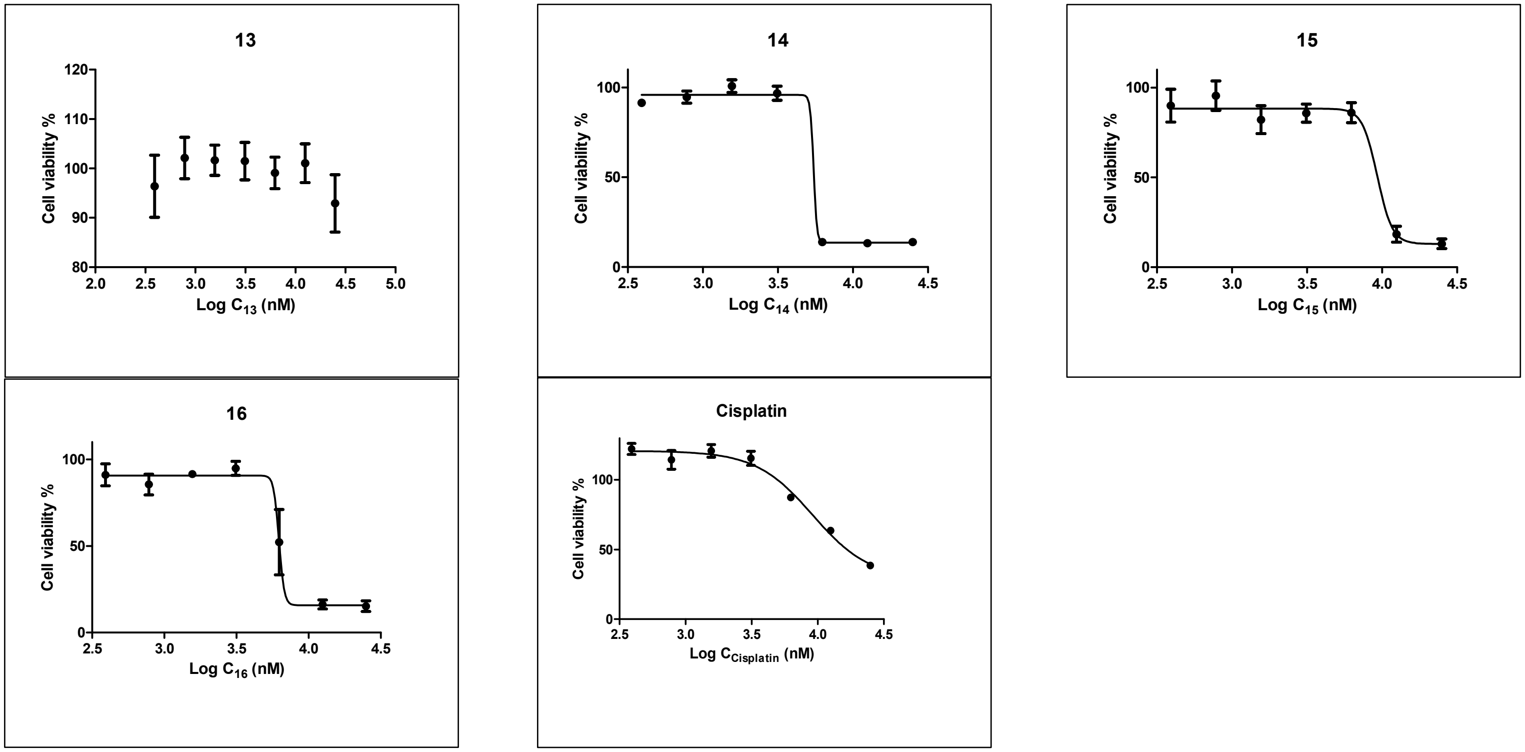

Figure S5. MTT assay was assessed on MDBK cells. The cells were seeded at 4000 cells per well. The MDBK cells were incubated with two- old serial dilution of compounds at dose range of 0.39 -25 μM for 5 days. Cell viability was determined using the standard MTT assay. Data was analysed using GraphPad prism. The experiments were performed three times; each concentration was repeated three times per experiment.

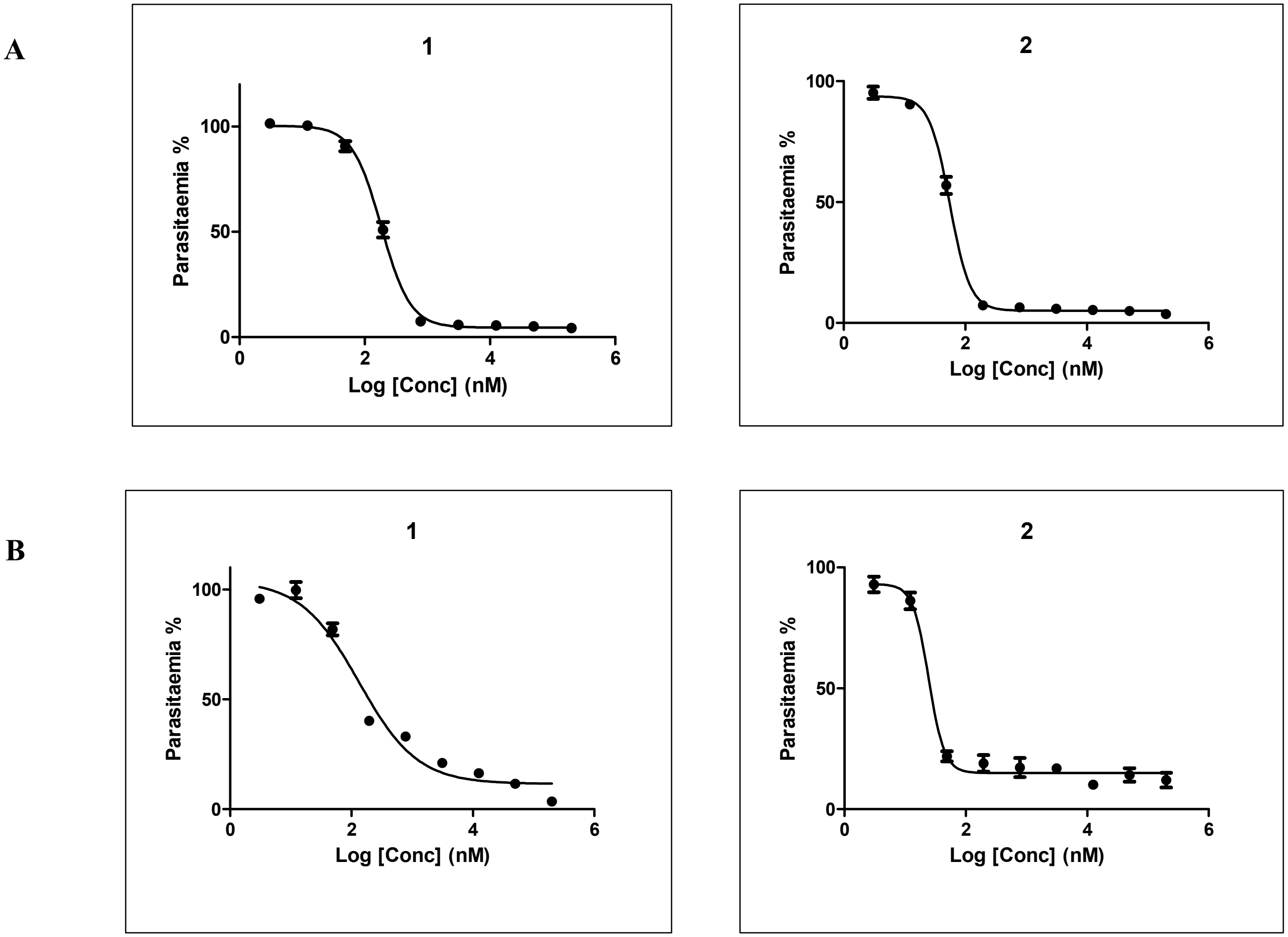

Figure S6. The effective dose of the lead 4-aminoquinoline hydrazone compounds **1** & **2** tested on the *P. falciparum* 3D7 A10 (A) and Dd2 (B) strains in a 4-fold serial dilution of dose range from 0.00305 - 200 μM. The compounds were incubated for 72 hours and read using SYBER green-based plate reader assay. The experiments were performed three times in triplicates. The data was analysed using GraphPad prism.

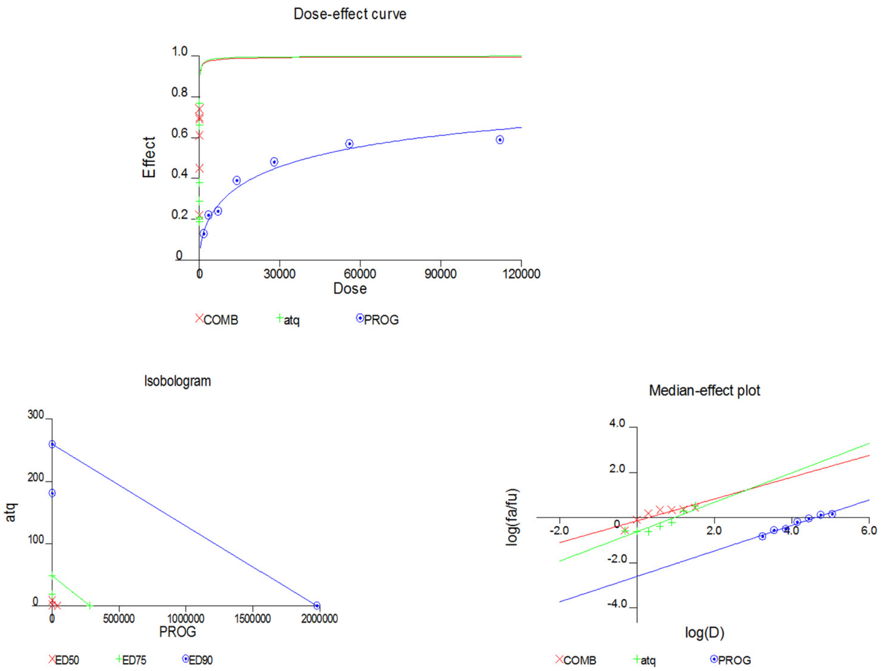

Figure S7. Dose effect analysis of atovaquone and proguanil combination on P. falciparum K1 strain. The dose-response data was analysed with CalcuSyn software to give a dose-effect curve and isobologram graph.

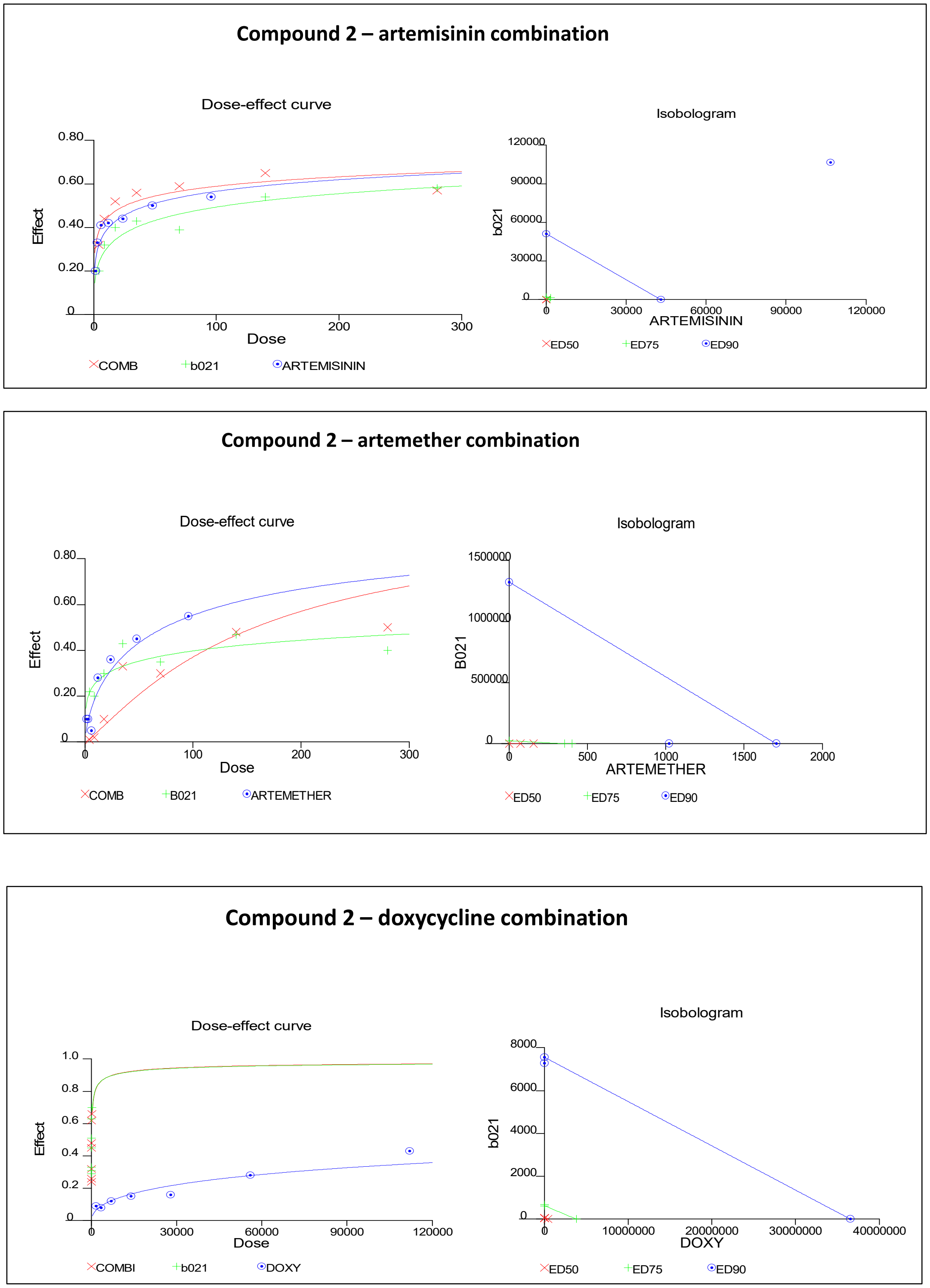

Figure S8: Dose effect analysis of Compound **2** and artemisinin, artemether and doxycycline combination on *P. falciparum* K1 strain. The dose-response data was analysed with CalcuSyn software to give dose-effect curve and isobologram graph.
